# Multiple system atrophy: an update and emerging directions of biomarkers and clinical trials

**DOI:** 10.1007/s00415-024-12269-5

**Published:** 2024-03-14

**Authors:** Min Liu, Zhiyao Wang, Huifang Shang

**Affiliations:** Department of Neurology, Laboratory of Neurodegenerative Disorders, Rare Disease Center, West China Hospital, Sichuan University, No. 37 Guoxue Xiang, Chengdu, 610041 Sichuan China

**Keywords:** Multiple system atrophy, α-Synuclein, Biomarkers, Disease-modifying therapies

## Abstract

Multiple system atrophy is a rare, debilitating, adult-onset neurodegenerative disorder that manifests clinically as a diverse combination of parkinsonism, cerebellar ataxia, and autonomic dysfunction. It is pathologically characterized by oligodendroglial cytoplasmic inclusions containing abnormally aggregated α-synuclein. According to the updated Movement Disorder Society diagnostic criteria for multiple system atrophy, the diagnosis of clinically established multiple system atrophy requires the manifestation of autonomic dysfunction in combination with poorly levo-dopa responsive parkinsonism and/or cerebellar syndrome. Although symptomatic management of multiple system atrophy can substantially improve quality of life, therapeutic benefits are often limited, ephemeral, and they fail to modify the disease progression and eradicate underlying causes. Consequently, effective breakthrough treatments that target the causes of disease are needed. Numerous preclinical and clinical studies are currently focusing on a set of hallmarks of neurodegenerative diseases to slow or halt the progression of multiple system atrophy: pathological protein aggregation, synaptic dysfunction, aberrant proteostasis, neuronal inflammation, and neuronal cell death. Meanwhile, specific biomarkers and measurements with higher specificity and sensitivity are being developed for the diagnosis of multiple system atrophy, particularly for early detection of the disease. More intriguingly, a growing number of new disease-modifying candidates, which can be used to design multi-targeted, personalized treatment in patients, are being investigated, notwithstanding the failure of most previous attempts.

## Introduction

Multiple system atrophy (MSA) is a rare, sporadic, and rapidly progressive neurodegenerative disorder characterized by progressive autonomic failure, parkinsonism, and cerebellar syndrome in various combinations [1]. Based on clinical symptoms, this neurodegenerative disorder can be classified into a Parkinsonian subtype (MSA-P) and a Cerebellar subtype (MSA-C). Macroscopic changes in MSA include selective atrophy of the striatonigral system and olivopontocerebellar system, which are presented by microscopic neuronal loss and axonal degeneration and correlated with the two clinical subtypes of MSA [2]. MSA is neuropathologically considered as one of the synucleinopathies due to the predominance of aggregated α-synuclein-positive cytoplasmic inclusions, classically localized in oligodendrocytes and termed glial cytoplasmic inclusions (GCIs) [3–5].

MSA is considered as the most aggressive synucleinopathy due to its rapid clinical course, typically leading to severe disability within 5–6 years and death on average within 10 years from symptom onset [6–9]. Although significant progress has been made in treating some symptoms of MSA, most MSA-related symptoms are not yet treatable with symptomatic therapies. In addition, most disease-modifying therapies have failed at the clinical trial stage, probably because pathologic changes were advanced when the disease was diagnosed. The new MSA diagnostic criteria developed by the Movement Disorder Society (MDS) aim to improve the diagnostic accuracy, particularly at the early stage [10, 11]. On the basis of the better understanding of the underlying pathophysiological mechanisms and improvement of early-stage diagnosis, the development of disease-modifying interventions for MSA has become an urgent unmet need.

This review summarizes the epidemiology and risk factors for MSA, neuropathologic progress, clinical presentations, updated diagnostic criteria, and treatment and clinical trials. More importantly, we provide a comprehensive and updated overview of current and emerging biomarkers as well as completed and ongoing clinical trials of disease-modifying therapies for this devastating disease.

## Epidemiology and genetics

MSA is a rare disease that could potentially affect the people of all racial groups without gender preference. According to a 10-year nationwide epidemiological study in Iceland, the incidence of MSA was estimated to be approximately 0.7 per 100,000 people [12]. According to a 15-year study conducted in Olmsted County, Minnesota, there were no one who developed MSA before age 50; however, the average annual incidence rate of MSA was 3.0 per 100,000 people [13]. In addition, studies from northern Sweden over a 4-year period and from Russia over a 2-year period found distinct frequencies of 2.1 and 0.1 per 100,000 people, respectively [14, 15]. This suggests that the incidence of MSA is influenced to some degree by region and age. Other studies estimate that the crude prevalence rates range from 1.9 in Gironde, 3.4 in Iceland, and 4.4 in London per 100,000 population [12, 16, 17].

In addition, the relative prevalence of MSA subtypes varies among geographical regions and ethnic groups. MSA-P is more prevalent in European and North American populations. MSA-C, on the other hand, is more common in Japanese and mestizo populations [7, 8, 18, 19].

MSA was traditionally considered as a sporadic disease rather than a genetic disease. However, there is increasing evidence from multiplex families with MSA, indicating genetic association in MSA [20, 21]. Genetic variants within *SNCA* locus (α-synuclein gene) were associated with an increased risk for the development of MSA [22]. There was strong genetic association between MSA-C subtype and *SNCA* replication [23], although the earlier study suggested that no nucleotide alterations were found in the entire region of *SNCA* in any of confirmed cases of MSA [24]. Four multiplex MSA families were studied using whole-genome sequencing. Two of the four Japanese families carried mutations in the coenzyme Q2 (*COQ2*) gene, which is involved in the coenzyme Q10 (CoQ10) synthesis pathway [25]. However, these results could not be replicated in a European cohort of patients with MSA [26, 27], suggesting that these variants may be population-specific. In addition, a common *COQ2* polymorphism *V393A* was associated with sporadic MSA in East Asia but not in the West [28–30]. In addition, other mutations of *COQ2*, such as *L402F*, tend to play a population-specific role in susceptibility to MSA in Chinese patients [31]. Other genetic risk factors, such as *C9orf72, LRRK2, MAPT*, etc., have also been reported [26]. A potential association between the family with the coexistence of MSA and amyotrophic lateral sclerosis (ALS) and hexanucleotide repeat expansions in *C9orf72* has been documented, but pathological confirmation would be needed to differentiate phenotypical presentations [32]. However, hexanucleotide repeat expansions in *C9orf72* were not found in a Chinese population with PD or MSA, indicating no associations between *C9orf72* expansions and the wider spectrum of Parkinsonism [33]. Pathogenic glucocerebrosidase (*GBA*) variants have been demonstrated to increase the risk of developing PD [34, 35]. A large-scale multicenter study identified *GBA* variants among MSA patients across the Japanese, European, and North American series, indicating *GBA* variants are associated with MSA [36]. Additionally, another study demonstrated that MSA patients from mainland China did not carry the *GBA L444P* mutation, but a large-scale study should be considered to further confirm the association [37]. In patients from the United States and the United Kingdom, exonic *LRRK2* variants have been found to be associated with MSA [38], but in the Han Chinese population, *LRRK2* variants were not risk factors for MSA [39]. However, another study reported an interesting result. One of eight subjects in the same family from the Sagamihara district with PARK8-linked parkinsonism (*LRRK 2 I2020T* mutation) had MSA pathology [40]. Additionally, another study of genome-wide association study (GWAS) identified four potential risk variants on genes of microtubule-associated protein tau (*MAPT*)*,* endothelin 1 (*EDN1*), f-box Protein 47 (*FBXO47*), and ELOVL fatty acid elongase 7 *(ELOVL7)* in MSA in European population [26], but no association was found in the Chinese population as presented in separate GWAS [41], suggesting that genetic risk factors for MSA maybe region- and ethnic-specific.

## Neuropathology

MSA-P and MSA-C correlate with neuropathological patterns of predominant striatonigral degeneration and olivopontocerebellar atrophy, respectively [42]. MSA belongs to the broad spectrum of α-synucleinopathies characterized by the abnormal accumulation of misfolded, hyperphosphorylated α-synuclein at serine residue 129 [43]. MSA is distinguished from other synucleinopathies by the presence of aggregated α-synuclein fibrils in oligodendrocytes (also known as GCIs) [3, 44]. Interestingly, multiple groups have found no evidence of elevated *SNCA* expression in MSA oligodendrocytes [45–47], indicating that *SNCA* gene expression is not the cause of MSA. In patients with MSA, α-synuclein fibrils accumulate more in oligodendrocytes but less frequently in the cytoplasm and nuclei of neurons, although α-synuclein is normally found mainly in neurons. The question of how α-synuclein was released from neurons and acted on oligodendrocytes in MSA has been challenged by the aforementioned feature. Increasing evidence suggests that pathological α-synuclein oligomers were released from neurons and may propagate α-synuclein pathology from neurons to oligodendrocytes, leading to neuronal loss and axonal degeneration in a specific brain region in a prion-like manner [48, 49]. However, the source of α-synuclein in GCIs in MSA brains remains obscure to date even though neuronal spreading appears to be a plausible source of it as described above. Additionally, oligodendroglial progenitor cells (OPCs) and immature oligodendrocytes express *SNCA* mRNA both in rodents and in humans. The density of OPCs was increased in a white matter region of the MSA brain, but α-synuclein does not accumulate in OPCs. It is still possible for OPCs to be mature oligodendroglia, which enables GCI formation [50, 51]. Another question is how α-synuclein oligomers were released and transported between different types of cells like neuron and glia. Exosomes are considered to be transporters of toxic α-synuclein oligomers. Both neurons and glial cells can release exosomes, which may contain inflammatory molecules and this glia-to-neuron or neuron-to-glia transmission of exosomal α-synuclein oligomers may contribute to the propagation of pathology and neuroinflammation throughout the brain in MSA [52].

**Fig. 1:**
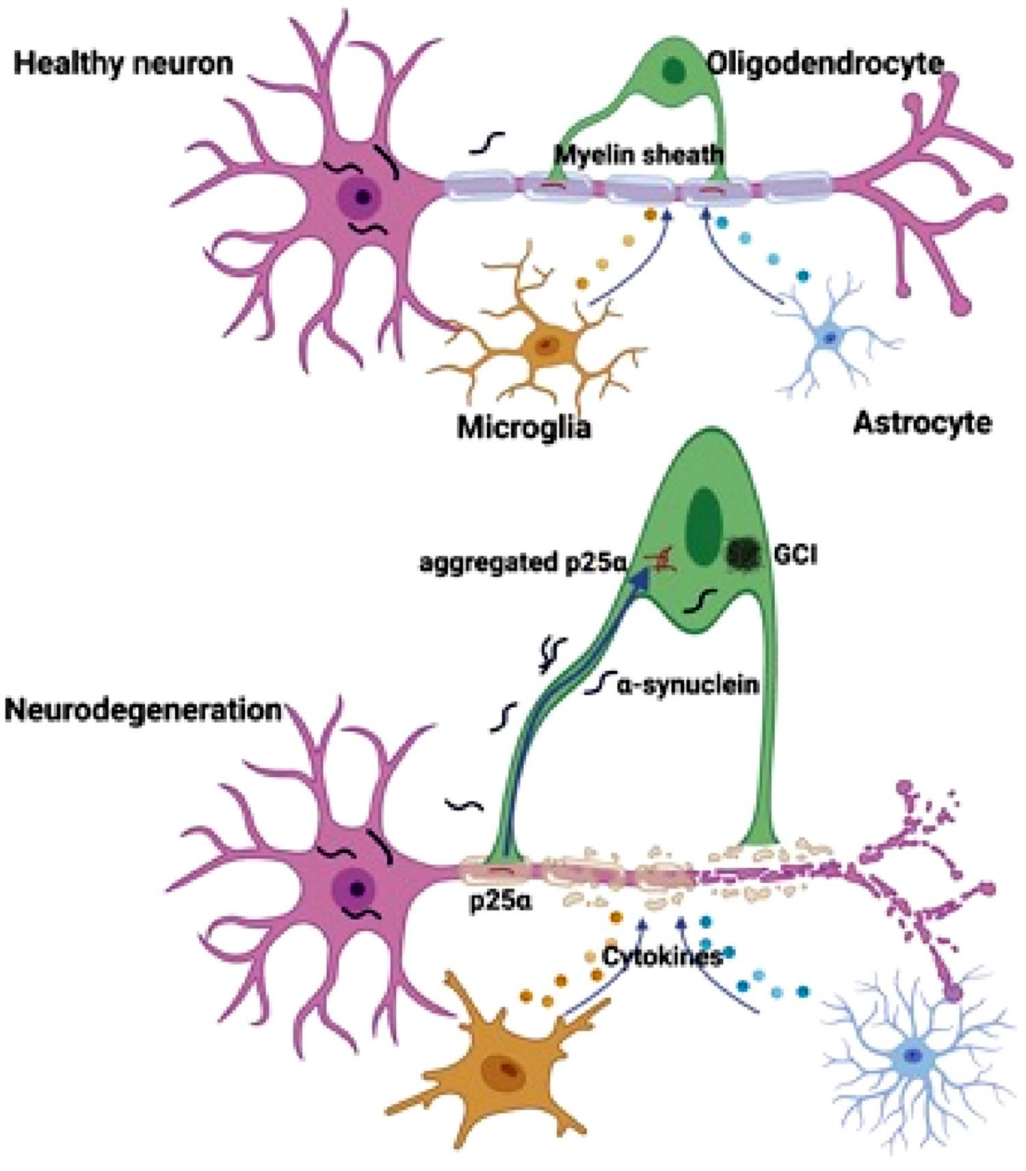
Key pathophysiological cascade events of MSA. The translocation of p25α from the myelin sheath to the oligodendroglial cell body, followed by p25α aggregation, appears to precede the abnormal aggregation of α-synuclein. This aggregated p25α led to morphological alteration and functional impairment of oligodendrocytes, rendering them unable to process the α-synuclein oligomer secreted by neurons, resulting in GCI formation and eventual neurodegeneration. Secondary events include astrogliosis and microglial overactivation. Created with Biorender.com and granted a publication license with Biorender (Agreement Number: GK255GQVD7)

α-Synuclein accumulates in oligodendrocytes exclusively in MSA, indicating a number of subcellular changes in oligodendroglia contribute to the pathogenic cascade and α-synuclein aggregation. In vitro studies suggested that phosphoprotein-25α (p25α, also known as tubulin polymerization promoting protein, TPPP) may be a potent trigger of α-synuclein aggregation. p25α, an oligodendrocyte-specific phosphoprotein, normally associates with the oligodendroglia marker myelin basic protein (MBP) located in the myelin sheath. An early-stage finding in patients with MSA revealed relocalization of p25α from the myelin sheath to the cell bodies, followed by accumulation of p25α in the cytosol and subsequent degradation and deposition of MBP in the cell bodies, and finally enlargement of oligodendroglia, which may lead to a decrease in cellular clearance [53–55]. The enlarged oligodendroglia were unable to degrade α-synuclein propagated from neurons [56]. Taking neuronal cytoplasmic inclusions (NCIs) reported in MSA [57] and dominant GCIs, as well as elevated astroglial and microglial activation together [58], eventually neuronal loss and axon degeneration occurred (Fig. 1). However, there are still a few key unanswered questions in the MSA pathogenic cascade, such as the timing of GCIs formation, the clear mechanism of interplay between neuron and glia, etc. [51].

## Clinical presentations

MSA is characterized by various combinations of autonomic dysfunction, parkinsonism, and ataxia, with the predominance of core motor symptoms defining MSA-P or MSA-C, although the symptoms of MSA-P and MSA-C often overlap [59]. Many patients have a mixed phenotype.

According to the current diagnostic criteria [10], prominent autonomic dysfunction (dysautonomia) is a critical defining feature in clinically established and probable MSA, with urogenital and cardiovascular systems primarily being affected and presenting in a variety of ways. Early and severe dysautonomia is indicative of a more aggressive disease course [60]. Core clinical features include neurogenic orthostatic hypotension (OH) and significant urinary dysfunction, such as voiding difficulty with retention after urination and urinary urge incontinence. Bladder dysfunction may be a prominent and common early symptom of MSA [61, 62]. Neurogenic OH for clinical established MSA is defined as a sustained drop in systolic blood pressure of  ≥ 20 mmHg or a drop in diastolic blood pressure of  ≥ 10 mmHg within 3 min of standing or head up tilt (HUT). In addition to recurrent syncope, other characteristic symptoms of OH include dizziness, nausea, weakness, tremors, headache, and painful sensations in the neck known as coat-hanger pain [63, 64].

In the past, the terms striatonigral degeneration and olivopontocerebellar atrophy were used to describe MSA, indicating the features of parkinsonism and cerebellar dysfunction. The core motor symptoms of MSA are parkinsonism and cerebellar ataxia, which correspond to MSA-P and MSA-C, respectively. Parkinsonism in MSA is dominated by a rapidly progressive poor levo-dopa responsive akinetic-rigid syndrome characterized by paucity and slowness of movement along with muscle stiffness and resistance to passive movements, unsatisfactory response to levo-dopa treatment due to striatal degeneration, and early postural instability and gait disability. Cerebellar syndrome include gait and limb ataxia, scanning dysarthria (also known as explosive speech), and cerebellar oculomotor disturbances, such as gaze nystagmus and hypometric saccades [65].

Patients with MSA also suffer from non-motor symptoms, such as cognitive deficits [66, 67], depression or anxiety [68, 69], sleep disturbances, and disordered breathing stridor [70, 71]. Rapid eye movement (REM) sleep behavior disorder (RBD), a parasomnia characterized by recurrent dream enactment with excessive motor behaviors such as punching or kicking, frequently affects patients with MSA. More than half of patients with MSA present with symptoms of RBD before the onset of motor deficits [72, 73]. In addition to sleep disturbance, laryngeal stridor is a diagnostic indicator of MSA with a high positive predictive value, and its early onset may contribute to shorter survival. *Stridor* is defined as a strained, high-frequency, harsh respiratory sound, primarily inspiratory, occurring only during sleep or both during sleep and wakefulness [74].

## Diagnosis and biomarkers

To date, three sets of MSA diagnostic criteria have been proposed for clinical and research purposes [10, 75, 76]. Over the past 14 years, the second consensus criteria have been widely used despite their suboptimal sensitivity [76–78]. Recently, a set of MDS MSA diagnostic criteria for clinical practice and inclusion criteria for clinical trials has been developed and validated against neuropathological diagnosis in current clinical practice [10, 79]. However, further validation studies are needed. The new criteria define four levels of diagnostic certainty: neuropathologically established MSA, clinically established MSA, clinically probable MSA, and possible prodromal MSA. They have incorporated current information and are anticipated to increase the sensitivity of future disease assessments.

Neuropathologically established MSA corresponds to the definite MSA category of the second consensus criteria. Autopsy must reveal widespread and abundant central nervous system (CNS) α-synuclein-positive GCIs along with neurodegenerative changes in striatonigral or olivopontocerebellar structures [10, 80]. Compared to the second consensus, the criteria for clinically established and probable MSA have been revised, including the mandatory value of MRI markers and a list of research biomarkers that were not previously required. Possible prodromal MSA is a research category, and future diagnostic biomarker research will expand this category. Identifying possible prodromal MSA at the earliest disease stage is crucial for being aware of fast progression and developing disease-modifying treatments for MSA. Possible prodromal diagnostic criteria were recently developed for MSA. Either polysomnography (PSG)-proven RBD or isolated autonomic failure (one of urogenital failure with post-void residual (PVR) > 100 ml or urinary urge incontinence, or neurogenic OH within 10 min of standing) are the current entry criteria for a diagnosis. Additionally, research biomarkers are similar across all the categories seen later in this review [10].

Autonomic dysfunction (Dysautonomia), parkinsonism, and cerebellar syndrome are still the essential clinical characteristics listed in the new criteria. Autonomic dysfunction has a significant impact on blood pressure and bladder control. The presence and severity of OH could be determined by cardiovascular autonomic function tests that measure supine and standing blood pressure and changes of heart rate. Continuous blood pressure monitoring, and HUT testing could provide additional information and help to differentiate MSA from similar diseases [81]. Although autonomic tests regarding PVR or HUT were performed easily and reported to be useful in the diagnosis of MSA, normal cardiac sympathetic imaging (^123^I-MIBG-scintigraphy) could also benefit the diagnosis of MSA [10] and even distinguishes PD from MSA patients. As cardiac sympathetic postganglionic denervation distinguishes PD from MSA patients with intact innervation, the radiolabeled noradrenaline analog ^123^I-MIBG may assist in distinguishing MSA from PD [82]. MSA patients exhibit preserved tracer uptake, whereas PD patients exhibit reduced tracer uptake. Furthermore, a supine plasma noradrenaline level > 100 pg/ml associated with neurogenic OH might support the diagnosis of MSA [83–85]. Taken together, imaging biomarker ^123^I-MIBG and plasma biomarker noradrenaline level maybe support the diagnosis of MSA before the appearance of severe autonomic dysfunction and motor disability. In addition, an elevated PVR volume (> 100 ml) is the most specific indicator of bladder impairment in MSA that can be detected by urodynamic testing or post-void bladder ultrasonography [86]. Poor or nonexistent response of parkinsonism to levo-dopa is a critical diagnostic feature for clinically established MSA. A poor levo-dopa responsiveness is typically defined by history or as < 30% improvement on the MDS-UPDRS III on up to 1000 mg levo-dopa as needed or tolerated for at least a month as judged by a movement disorder specialist [10].

**Fig. 2:**
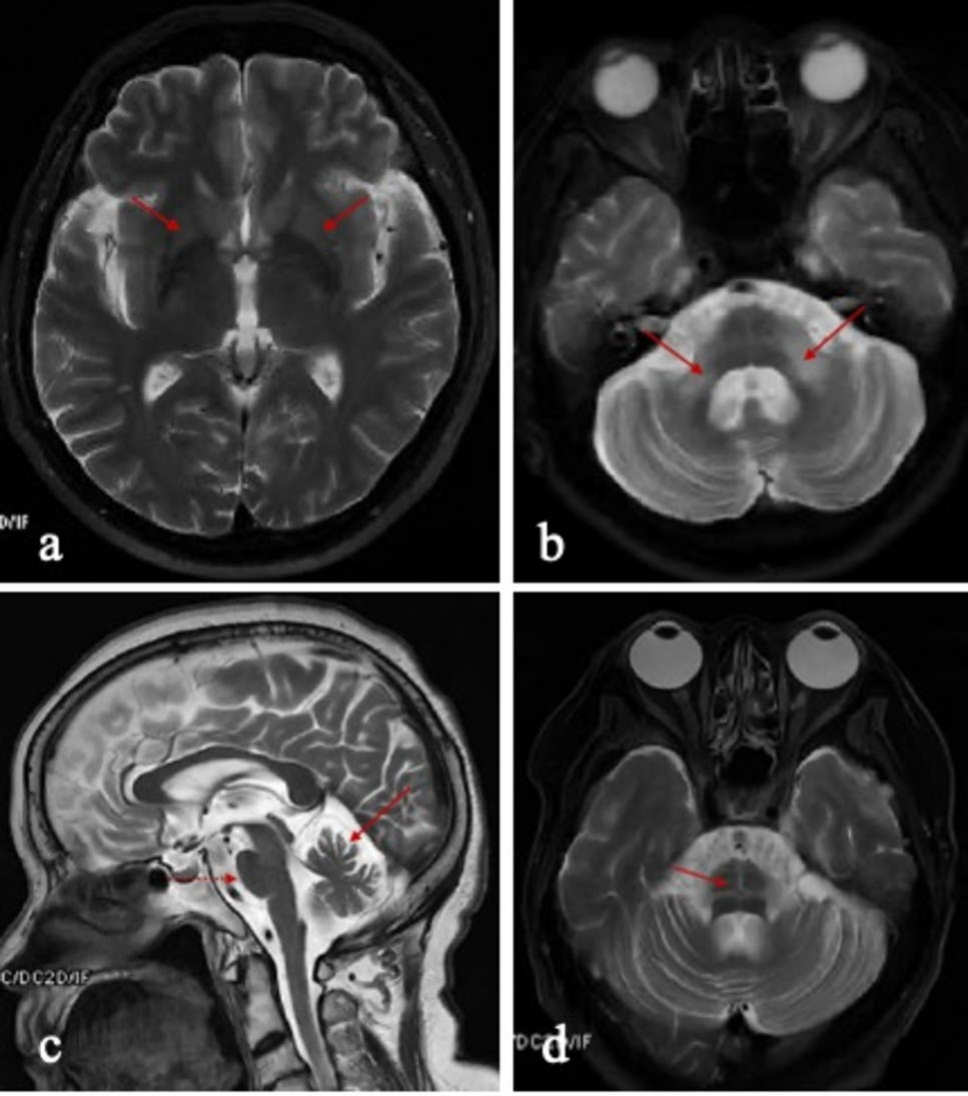
MRI brain imaging in MSA patients. **a** Demonstrates putaminal atrophy (red arrows). **b** Atrophy and hyper-intensity signal of middle cerebellar peduncles (red arrows). **c** Pontine atrophy (red dashed arrow) and cerebellar atrophy (red solid arrow). **d** “Hot cross bun” sign (red arrow)

According to the new MSA diagnostic criteria, imaging evidence is required for clinically established MSA (Fig. 2). Regarding neuroimaging, radiotracer-based functional imaging techniques could support the diagnosis of MSA. In MSA-C, olivopontocerebellar atrophy predominates, with loss of pontocerebellar fibers and gliosis which is best seen on T2-weighted MRI imaging, occasionally producing the characteristic "hot cross bun" sign [87]. In MSA-P, MRI may reveal putaminal atrophy with decreased T2 signal in the posterior putamen [88]. In addition to MRI, ^18^FDG- positron emission tomography (PET) has been shown to be helpful in diagnosing MSA by revealing hypometabolism in the putamen, pons, and cerebellum [89]. Currently, some efforts are being made in the development of selective α-synuclein PET tracers, despite numerous obstacles in visualizing intracellular α-synuclein inclusions in synucleinopathies, including intracellular localization α-synuclein, low abundance of α-synuclein within brain [90, 91]. Hopefully, this non-invasive in vivo imaging technique can benefit the diagnosis of MSA in the future.

Although detection of MSA biomarkers in body fluids or tissues is not required in diagnostic criteria and is still under investigation, promising molecular markers have been summarized and utilized in preclinical and clinical studies of MSA [92–94]. Several investigational biomarkers, including α-synuclein, neurofilament light chain (Nfl), and total tau, have demonstrated potential value but have not been routinely used because of their limited availability and lack of diagnostic validation. A meta-analysis suggests that a combination of a decrease in α-synuclein and total tau and an increase in Nfl in cerebrospinal fluid (CSF) distinguishes MSA patients from healthy controls [95]. In addition, a recent pilot study showed that the levels of phosphorylated α-synuclein at serine 129 (pS-α-syn) were significantly higher in MSA patients than in healthy controls, suggesting that phosphorylated α-synuclein in red blood cells is a potential diagnostic biomarker for MSA [96]. Excluding plasma, peripheral tissues, such as mucosa, salivary glands, and skin, were examined to develop a safer and convenient method for supporting MSA diagnosis. Skin biopsy to detect α-synuclein has demonstrated biomarker potential [97]. Moreover, seeding aggregation assays, including Protein Misfolding Cyclic Amplification (PMCA) and Real-Time Quaking-induced Conversion (RT-QuIC), have been developed to detect pathological α-synuclein in CSF with high sensitivity and specificity [98–100]. One study reported that α-synuclein was not only detected by PMCA with high sensitivity in the CSF of both PD and MSA, but also had distinguished amplification kinetics [99]. Meanwhile, the samples of skin, olfactory mucosa, and submandibular gland were also seeded with α-synuclein. The seeding of α-synuclein aggregation in skin provided evidence that α-synuclein in skin can serve as a novel biomarker for synucleinopathies [101]. Another study suggests that the combination of skin α-synuclein RT-QuIC and circulating Nfl can distinguish between MSA and PD [102]. In addition, a few studies utilizing the RT-QuIC assay demonstrated the potential to detect α-synuclein aggregation in other peripheral tissues, including the olfactory mucosa and submandibular gland [103, 104]. Phosphorylated α-syn in non-myelinating Schwann glial cells of the skin has been studied and may be a promising clinical biomarker for MSA [105, 106].

Importantly, recent studies have also focused on measuring oligomer α-synuclein in the exosomes as another non-surgical approach for the diagnosis of MSA. Exosomes are one main subtype of extracellular vesicles (EVs) formed by an endosomal route, and are secreted by all cell types, including neuron, oligodendroglia and have been found in plasma, CSF, saliva and other secretions [107]. Exosomes have been shown to carry various proteins, including α-synuclein [52]. So, this indicates that exosomes in the blood that have originated from various brain cells could reflect pathogenic status. To identify blood-based biomarkers in MSA, neuron- and oligodendroglia-derived exosomes containing α-synuclein appear promising and feasible. One study showed that the concentration of α-synuclein in the oligodendroglia-derived exosomes in the plasma of MSA patients was lower as compared to the healthy controls, but it remained the same in the neuron-derived exosomes [108]. However, in contrast to the above study, another study presented conflicting data. They identified elevated concentrations of α-synuclein within both neuron- and oligodendroglia-derived exosomes in the blood of MSA patients compared to the healthy controls [109]. Despite different studies showing conflicting results, exosomal contents may serve as non-surgical and safe MSA biomarker if further efforts of optimization and validation can be conducted.

In addition, with the support of current diagnosis criteria including typical clinical manifestations and tests, imaging and fluid biomarkers, and more sensitive and accurate measurements like PMCA and RT-QuIC as well, the earlier and more reliable diagnosis of MSA became possible and more convincing. However, there was some combination of PD and MSA in autopsy level in MSA patients [110], which made the diagnosis more difficult. Meanwhile, as MSA belongs α-synucleinopathy, which included PD, dementia with Lewy bodies and MSA, Lewy bodies were present in about 10–13% of MSA cases [111, 112]. Overlap of clinical or pathological presentation made a clear and solid diagnosis more challenging. Therefore, more sensitive and specific measurements are urgent to develop to differentiate the diseases with common manifestations and avoid misdiagnosis. And the development of broader biomarkers at the early stage maybe could help the diagnosis.

## Treatment and clinical trials

Current treatment focuses primarily on symptomatic management [113–115]. This review only summarized the clinical trials of disease-modifying therapies. Disease modification could be defined as interventions that influence the underlying pathophysiology of MSA and have a positive outcome on disease progression.

Over the past two decades, the number of clinical trials to modify MSA has increased. Unfortunately, most attempts have failed, possibly due to incomplete understanding of pathophysiology, inadequacies in preclinical animal models, and lack of early and accurate diagnosis of the disease.

The most straightforward therapeutic target and treatment strategy is directed against pathological oligomer α-synuclein. Other strategies aim to enhance the synaptic function, restore the proteostasis, inhibit neuroinflammation and provide neuroprotection for neuronal death [116]. Table 1 provides an overview of previous and current studies on the development of disease-modifying therapies for MSA.

**Table 1:** Summary of Therapeutic Clinical Trials

Hallmarks	Mode of action	Investigational drug	Phase	Primary outcome	Results and Current Status
Pathological protein aggregation	Active immunization against α-syn	AFFITOPE PD01A, PD03A	1	Safety and Tolerability	Safe and well-tolerated
	Passive immunization against α-syn	Lu AF82422	1, 2	Safety and Tolerability and Efficacy	Safe and well-tolerated
	Passive immunization against α-syn	PRX002, MEDI1341, BIIB054	1, 2	Safety and Tolerability and Efficacy	In PD
	Passive immunization against α-syn	Tak-341	2	Safety and Tolerability and Pharmacokinetics	Ongoing
	Antisense oligonucleotides	BIIB101 (ION464)	1	Safety and Tolerability and Pharmacokinetics	Ongoing
	Inhibition of α-syn aggregation	EGCG	3	UMSARS Part II Score	Negative
	Inhibition of α-syn misfolding	NPT200-11	2	UPDRS, Parts I-III Score	In PD
	Inhibition of α-syn aggregation	Anle 138b	1	Safety and Tolerability	Safe and well-tolerated, Phase 1b in PD
	Inhibition of α-syn aggregation	ATH434 (PBT434)	2	Safety and Efficacy	Ongoing
Synaptic dysfunction	Synaptic density	N/A	N/A	N/A	Observational
Aberrant proteostasis	Enhanced degradation of α-syn	Sirolimus (rapamycin)	2	Efficacy (A Futility Trial)	Terminated
	Enhanced degradation of α-syn	Rifampicin	3	UMSARS Part I Score	Terminated
	Enhanced degradation of α-syn	Lithium	2	Safety	Terminated
**Neuroinflammation**	Inhibition of microglial activity	Minocycline	3	UMSARS Part II Score	Negative
	Inhibition of inflammation	IVIG	2	Number of the adverse effects	Positive
	Inhibition of oxidative stress	Verdiperstat (AZD3241, BHV-3241)	3	Standardized uptake values and ratios	Negative
Neuronal cell death	Reduced excitotoxicity	Riluzole	3	UMSARS Part II and III Score	Negative
	NMDAR-modulator	Tllsh2910	3	SARA score	Ongoing
	FAF-1 inhibition	KM-819	1	Safety and Tolerability	Safe and well-tolerated
	Lipidomic neurotoxicity	YTX-7739	1b	Safety and Tolerability	Safe and well-tolerated
	Mito dysfunction	CoQ10	2	UMSARS Part II Score	Ongoing
	Proliferation	Growth hormone	2	UMSARS total score	Ongoing
	Mito dysfunction	Rasagiline	2	UMSARS Part I and II Score	Negative
	Mito dysfunction and reducing glutamate release	Safinamide	2	TEAEs and SAEs	Ongoing
	Neurotrophic support	Fluoxetine	2	UMSARS Part IandII Score	Negative
	Neuroprotection	GDNF	1	Safety and Tolerability	Safe and well-tolerated
	Neuroprotection	Insulin	2	UPDRS-Motor	Motor improvement in PD
	IGF-1	Exendin-4	2	UMSARS Part I and II Score	Ongoing
	Neuroprotection	ONO-2808	2	Safety, Tolerability, Pharmacokinetics, Pharmacodynamics, and potential efficacy	Ongoing
	Neuroprotection	bmMSCs	2	UMSARS Part II Score	Positive
		fMSCs	1/2	Safety and Tolerability	Safe and well-tolerated
		hOMSCs	1/2a	Safety and Efficacy	Ongoing

### Targeting pathological α-synuclein

MSA is characterized by the accumulation of insoluble oligomer α-synuclein within oligodendrocytes, also referred as GCIs. Although there is no direct evidence that α-synuclein is the cause of MSA progression, an abundance of evidence supports the potential significance of α-synuclein to the pathogenic cascade of MSA [51]. Treatment strategies targeting α-synuclein have been widely adopted and evaluated in clinical trials. These include immunotherapy, inhibition of α-synuclein aggregation, and α-synuclein gene therapy by antisense oligonucleotides (ASO) [116]. Due to the pathological similarity between PD and MSA and the fact that PD is the most prevalent synucleinopathy, several potential disease-modifying treatments have been investigated in both PD and MSA patients. In addition to MSA, we list the relevant clinical trials from PD in which the same above strategies were used, although these treatments are not currently available in MSA.

#### Immunotherapy of α-synuclein

In recent years, increasing evidence has supported the theory that α-synuclein is primarily produced by the neurons, where it aggregates and spreads into oligodendrocytes via the extracellular environment, as mentioned earlier. This provided a clear rationale for targeting the α-synuclein-mediated extracellular pathology through immunotherapeutic approaches. Both active and passive immunizations targeting the reduction of α-synuclein are promising immunotherapeutic modalities currently under development.

MBP-α-synuclein transgenic mice, a mouse model of MSA in which α-synuclein is specifically expressed in oligodendrocytes, were used to investigate the active immunization of α-synuclein. In the study, immunization with AFFITOPE® (AFF 1) reduced the accumulation of α-synuclein within oligodendrocytes, prevented demyelination, and ameliorated motor deficits and neurodegeneration in the above mouse model. Moreover, the increase in α-syn-positive microglial cells suggests that immunization with AFF 1 stimulates the degradation of α-synuclein by microglia [117]. Subsequently, to evaluate the safety and tolerability of AFFITOPE® PD01A in patients with PD, a first-in-human phase 1 immunization trial showed that it was safe and well tolerated [118] (NCT01568099, NCT02216188). Another phase 1 study evaluating the safety and exploring the immunogenicity of AFFITOPE® PD01A and PD03A in patients with early MSA demonstrated that both PD01A and PD03A were safe and well tolerated and that PD01A elicited a rapid and long-lasting antibody response [119] (NCT02270489). Further large-scale studies are needed to determine the efficacy of vaccines if they can slow the progression of MSA.

In addition to the active immunization strategy mentioned above, some treatments based on passive immunization are currently being developed. Lu AF82422 from Lundbeck is a human monoclonal antibody (mAb) directed against the toxic α-synuclein protein. A phase 1 interventional study evaluating the safety and tolerability of the antibody in healthy volunteers and in Parkinson's disease (PD) has shown that it is safe and well tolerated (NCT03611569). Lundbeck has recently advanced the development of Lu AF82422 in MSA with the AMULET study, a phase 2 trial to evaluate safety, tolerability, and efficacy in MSA. This study has been enrolling patients since June 2022 (NCT05104476).

Other passive immunization-based treatments are currently being investigated only in PD and not in MSA patients. Given that the mechanism of action of these therapies is directed against α-synuclein, they are likely beneficial to MSA treatment. One of such therapies is Prasinezumab (PRX002) from Prothena/Roche. A first-in-human trial in which PRX002 was administered by intravenous infusion in PD patients revealed to be safe and well tolerated. And the pharmacokinetic properties of the drug showed strong binding of peripheral α-synuclein and a dose-dependent increase of PRX002 in CSF [120, 121] (NCT02157714). However, in a subsequent large-scale phase 2 study (PASADENA), the primary endpoint of slowing disease symptom progression in PD patients over one year, as measured by change in MDS-UPDRS-total score, was not met [122]. Nonetheless, interestingly, PRX002 showed signals of efficacy on secondary measures, significantly delaying worsening of the UPDRS Part III, i.e., digital motor score, by 25–30%, and patients with more severe and rapidly developing symptoms appeared to have benefited most from treatment (NCT03100149). Based on the research from PASADENA, Roche initiated another phase 2b study (PADOVA) in patients with more advanced symptoms than in the PASADENA study to further evaluate the efficacy and safety of PRX002, refining the primary endpoint to time to meaningful disease progression to MDS-UPDRS part III (NCT04777331). Another α-synuclein antibody, MEDI1314, is being developed by AstraZeneca. Two phase 1 studies evaluating the safety and tolerability, pharmacokinetics, and pharmacodynamics of MEDI1341 in healthy volunteers and PD patients, respectively, have been completed (NCT03272165 and NCT04449484). Currently, Takeda took over its development (Tak-341) to evaluate the efficacy, safety, tolerability, pharmacokinetics, and pharmacodynamics of intravenous (IV) TAK-341 in participants with MSA in phase 2 stage (NCT05526391).

The fourth monoclonal antibody in clinical trials is Biogen's BIIB054 (Cinpanemab). SPARK, a phase 2 trial of BIIB054 in PD patients has been terminated since it had failed to meet its primary and secondary endpoints, despite phase 1 results showed it was safe and well tolerated [123] (NCT02459886, NCT03318523).

#### Antisense oligonucleotides

As a method to modify gene or protein expression, antisense oligonucleotides (ASO) are increasingly being explored clinically to target some causative genes associated with multiple neurologic diseases, such as Alzheimer's Disease (AD), spinal muscular atrophy (SMA), and Huntington's Disease (HD) and non-neurologic diseases, such as Duchenne muscular dystrophy (DMD). Specifically, Food and Drug Administration (FDA) approved the ASO drug Nusinersen (Spinraza) for the treatment of SMA, and it recently approved another ASO drug Casimersen (Amondys 45) for the treatment of DMD [124, 125]. In addition, the findings of a recent phase 3 trial evaluating the efficacy and safety of intrathecally delivered ASO medication RO7234292 (RG6042) in HD patients are awaited (NCT03761849). The development outlined above suggests that ASO is likely to become a significant new therapeutic category for neurodegenerative diseases and rare diseases.

Given that intracellular aggregation of α-synuclein plays a critical role in the MSA pathology, reducing the α-synuclein protein production using ASO targeting *SNCA* gene is a straightforward and promising mechanism of action, although current research in this area remains at an early stage. ASO of α-synuclein has shown neuroprotective effects and a dramatic reduction of α-synuclein in CSF and brain tissue in the PD animal models [126]. A phase 1 first-in-human trial with intrathecal application of the ASO ION464 (previously known as BIIB101) in MSA patients is ongoing (NCT04165486). This study is to evaluate the safety, tolerability, pharmacokinetics, and pharmacodynamics of ION464.

Synucleinopathies are generally characterized as gain of function in *SNCA*. Indeed, ASO-mediated suppression of α-synuclein reduced α-synuclein pathology in a dose-dependent manner. α-synuclein pathology will be rescued if the *SNCA* mRNA levels is allowed to return to normal levels [127]. However, an excessive α-synuclein knock-down may have detrimental effects. For example, a reduction of dopamine content in the striatum and tyrosine hydroxylase (TH) -positive neurons in the substantia nigra pars compacta (SNpc) were revealed in α-synuclein knockout mice [128, 129]. Thus, an appropriate level of α-synuclein achieved by ASO of α-synuclein is crucial. In addition, non-invasive and efficient delivery of ASO drug candidate may be another intractable aspect to overcome in this field.

#### Inhibition of α-synuclein aggregation

As previously stated, α-synuclein-positive GCI is the pathological hallmark of MSA, nevertheless the absence of endogenous oligodendroglial α-synuclein expression obscured the pathological detail. Targeting the misfolding and aggregation of α-synuclein has always been one of the major treatment strategies for MSA.

Epigallocatechin gallate (EGCG), a small molecule extract of green tea, inhibited the fibrillogenesis of α-synuclein by directly binding to the natively unfolded α-synuclein polypeptide chain and preventing its conversion into toxic aggregation forms, thereby reducing associated toxicity [130, 131]. Although EGCG inhibited α-synuclein aggregation in vitro and alleviated motor impairments and α-synuclein aggregation in nonhuman primates [132, 133], a phase 3 trial in patients with MSA failed to alter the disease but revealed overall well-tolerated [134] (NCT02008721).

Another small molecule, NPT200-11 (also knowns as UCB0599), has also demonstrated α-synuclein modulation-related benefits in animal models of PD [135, 136]. A phase 1 trial in healthy subjects determined the safety, tolerability, and blood levels of orally administered NPT200-11 (NCT02606682), and a phase 2 trial in patients with early PD and mild symptoms is currently underway (NCT05543252).

Anle138b is a small molecule that targets and inhibits α-synuclein oligomerization and aggregation in SNpc and striatum in MSA mouse models, slowing the progression of the disease [137–139]. The German biotech company MODAG successfully completed a phase 1 trial in which Anle138b demonstrated an excellent safety and tolerability profile (NCT04208152). Currently, another phase 1 trial is evaluating the safety, tolerability, blood levels, and efficacy of orally administered anle138b in patients with mild to moderate PD (NCT04685265).

In addition, dysregulation of iron metabolism was a potential pathogenic factor in neurodegenerative diseases. It has been shown that iron was elevated in PD and MSA [140, 141]. Iron could regulate α-synuclein expression at the translational level and mediate the aggregation of α-synuclein [142]. PBT434 (also known as ATH434) was a novel small molecule inhibitor of α-synuclein aggregation by redistributing excessive loosely bound iron [143–145]. Accordingly, a phase 1 trial of it was completed, demonstrating that it was safe, well-tolerated, and achieved the desired brain concentration in healthy subjects [146] (U1111-1211-0052). Currently, a phase 2 trial is evaluating the safety and efficacy of ATH434 in patients with MSA (NCT05109091, NCT05864365).

### Synaptic dysfunction

In neurodegenerative diseases, synaptic dysfunction and toxicity seem to be an early event preceding neuronal loss. α-synuclein exerts its physiological function in the synapses, thereby it is plausible that MSA may be characterized by extensive synaptic loss [147]. A recent observational in vivo study regarding the degree of damage of the synapses using the synaptic vesicle glycoprotein 2A (SV2A) selective PET radioligand [^11^C] UCB-J showed a profound loss of synaptic density in the putamen, cerebellum, and brainstem (NCT05121012). This study will show how early the synapse deteriorates in MSA patients and open new avenues for its treatment preceding the significant neuronal loss and clinical symptoms.

### Aberrant proteostasis

The impairment of protein processing and degradation has been implicated in the pathogenesis of α-synuclein aggregation based on some preclinical evidence [148–150]. Therefore, enhancing the degradation of α-synuclein has also been adopted as one important treatment strategy for MSA.

Accumulating evidence suggests that the autophagy–lysosomal pathway is altered in MSA. By inhibiting the activity of mammalian target of rapamycin (mTOR), rapamycin (also known as Sirolimus) influenced a variety of essential cellular processes, such as protein synthesis and autophagy [151, 152]. Rapamycin increased autophagy, decreased α-synuclein aggregation and provided partial neuroprotection in the SNpc of PLP-α-syn transgenic mice (PLP: proteolipid protein), a specific MSA mouse model [153, 154]. However, a phase 2 trial on oral sirolimus for MSA was failed recently (NCT03589976).

Another degradation enhancer is rifampicin, an antibiotic which has been investigated to inhibit the α-synuclein fibrils and to disaggregate already-formed fibril [155]. In addition, rifampicin decreased α-synuclein and neurodegeneration in MBP-α-syn transgenic mouse model of MSA [156]. A large phase 3 trial in patients with early MSA was terminated after an interim analysis of the primary endpoint revealed that futility criteria were met (NCT01287221).

Besides, lithium exerted neuroprotection against rotenone-induced injuries partially through the autophagy pathway in an in vitro PD cell model [157] and protected dopaminergic neurons likely via autophagy enhancement in an MPTP-administration mouse model of PD [158], indicating that it may be beneficial in MSA. A phase 2 trial to evaluate efficacy, safety, and tolerability of lithium treatment in patients with MSA was terminated due to severe adverse effect from interim analysis, indicating that lithium treatment was not well-tolerated [159] (NCT00997672).

Despite the failure of all clinical trials focusing on α-synuclein degradation enhancement to slow down the progression of the disease, several promising preclinical studies are still underway. On the other hand, it has been demonstrated that α-synuclein can be degraded via the autophagy and the ubiquitin–proteasome system (UPS) pathways [160]. Targeting the proteasome degradation system may be a viable alternative, as suggested by the failure of clinical trials aimed at enhancing the autophagy-lysosomal pathway. Recently, by introducing the proteolysis targeting chimeric (PROTAC) concept and technology, a peptide fusion containing α-synuclein binding domain and a short strong proteasome-targeting motif was able to bind to α-synuclein and direct it to the proteasome for degradation [161]. Additionally, one of Arvinas Company’s pipelines demonstrated that α-synuclein PROTAC protein degraders could degrade oligomeric forms of α-synuclein in their preclinical studies.

### Inhibition of neuroinflammation

There was growing evidence that brain inflammation played a crucial role in the pathogenesis of MSA. In MSA, aggregated α-synuclein induced microglial activation and astrogliosis, stimulated the secretion of proinflammatory cytokines in microglia, and ultimately exacerbated the disease pathology [58, 162, 163]. Therefore, suppression of microglial activation or inflammation as a whole has been viewed as a potential and promising approach in MSA.

Minocycline was a semi-synthetic, second-generation tetracycline analog which can effectively cross the blood–brain barrier and inhibit the microglial activation [164, 165]. Notably, minocycline has been shown to provide neuroprotection in experimental models of multiple neurodegenerative diseases, including PD, Alzheimer’s Disease (AD) and HD [166–168]. Contradictory preclinical evidence suggested that minocycline did not prevent lesion-induced neuronal damage in a rat model of striatonigral degeneration, a core pathology that may correlate with MSA, despite its microglial suppression [169]. In addition, another study found that minocycline inhibited microglial activation, but exacerbated dopaminergic neuronal damage in a mouse model of PD administered with MPTP [170]. A phase 3 for the evaluation of the efficacy and safety of Minocycline for treatment of MSA was completed (NCT00146809) but failed to demonstrate a clinical effect of minocycline on symptom severity as measured by clinical motor function. However, preliminary PET data suggested that minocycline may inhibit microglial activation [171].

Another study investigated the intravenous administration of immunoglobulins (IVIG). IVIG is a type of antibody mixture derived from human plasma that is believed to inhibit auto-reactive T-cells and then the production of cytokines. Even though the underlying mechanism remains poorly understood, this is utilized in a variety of immune-mediated neurological diseases. Activation of microglia and production of toxic cytokines suggested a role for neuroinflammation [172] and the potential therapeutic benefit of IVIG in MSA. A phase 2, open-label pilot clinical study for the efficacy of IVIG had enrolled 9 MSA patients, and the UMSARS scores declined in the majority of this group of patients [173] (NCT00750867). Despite this, to further confirm the efficacy of IVIG, larger confirmatory trials are still needed.

In addition, myeloperoxidase (MPO), a key enzyme involved in the production of reactive oxygen species by phagocytic cells, contributed to the oxidative stress implicated in the pathogenesis of the neurodegenerative disorders, such as AD, PD, multiple sclerosis, and MSA [174–177]. In the MSA mouse model, it was demonstrated that MPO inhibition reduced motor impairment and rescued vulnerable neurons in the striatum, SNpc, cerebellar cortex, pontine nuclei, and inferior olives, which was accompanied by a reduction in microglial activation and intracellular aggregates of α-synuclein [177]. In contrast, another research reported that MPO inhibition had no effect on motor impairments and neuronal loss in a mouse model of advanced MSA despite a significant decrease in microglial activation [178]. Verdiperstat (also known as AZD3241, and BHV-3241) is a potent, selective, and irreversible inhibitor of MPO that suppresses microglial activation that was initially studied by AstraZeneca [179]. A phase 1 study evaluating the safety of AZD3241 in healthy participants revealed this compound has a good safety profile (NCT01457807). A phase 2 PET study in PD demonstrated that AZD3241 was safe and well tolerated and reported amelioration of microglial activation, supporting proof of the mechanism of AZD3241 and extending further study of AZD3241 in PD or MSA [180] (NCT01527695). A phase 2 12-week trial of AZD3241 clinical trial in patient with MSA to assess the effect on microglia activation as measured by PET using 11-^C^PBR28 tracer, a tracer that binds to the transporter protein (TSPO) in activated glia as primary outcome was completed and no significant changes from baseline, or between groups, were detected (NCT02388295). In 2018, Biohaven licensed AZD3241 from AstraZeneca and further developed by a phase 3 clinical trial of BHV-3241 (referred to AZD3241) in patients with MSA, given the supportive phase 2 exploratory efficacy outcomes. However, this phase 3 study to evaluate the efficacy and safety of BHV-3241 in subjects with MSA was recently completed and failed to meet its primary and key secondary endpoints (NCT03952806). Recently, Biohaven began a small study evaluating the newer TSPO PET ligand 18^F^PBR06, before and after Verdiperstat treatment [181]. This study enrolled 19 MSA, studied the effect of Verdiperstat on microglial activation in well-characterized MSA patients and was completed in January 2022 (NCT04616456).

### Neuroprotective therapies

It is well-known that glutamate-induced neurotoxicity plays a crucial role in the neuronal damage and death underlying a broad spectrum of central nervous system disorders. Several glutamate receptor antagonists have been investigated for their neuroprotective effect in CNS disorders. Riluzole, an anti-glutamatergic agent approved by the FDA as a disease-modifying therapy for amyotrophic lateral sclerosis (ALS), is believed to have neuroprotective properties [182]. Observations of a reduction in behavioral deficits and striatal degeneration in the double lesion rat model of MSA-P administered by Riluzole supported the neuroprotective effect [183]. Despite promising preclinical results, a placebo-controlled cross-over trial in 10 probable MSA patients showed no significant anti-parkinsonian effects after administrated Riluzole [184]. A subsequent, large-scale phase 3 trial of Riluzole in MSA (NNIPPS) failed to meet the primary endpoint which is the difference of 36-month survival rate between the placebo group and the treatment group [185] (NCT00211224). Other neuronal excitability modulators are currently under investigation, such as Tllsh-2910, a specific NMDA receptor modulator. NMDA receptors in the cerebellum have unique properties that distinguish their function and modulation from those in other brain regions [186]. A phase 3 clinical trial of Tllsh-2910 in patients with MSA is ongoing and its primary endpoint is the improvement rating of ataxia (NCT03901638).

Fas-associated factor 1 (FAF1) is an apoptosis-related Fas-binding protein. It has been reported that FAF1 levels were significantly elevated in PD and were responsible for neuronal cell death [187, 188]. In addition, FAF1 induced α-synuclein accumulation in dopaminergic neurons [189]. KM-819 is a novel FAF1 inhibitor and could act as a neuroprotective agent. The effect of KM-819 in dopaminergic neurons of MPTP mouse model of PD was investigated. The study manifested the neurorestorative effect of KM-819 in striatal dopamine neurons of MPTP model via restoring autophagic α-synuclein degradation [190, 191], implying that KM-819 may have therapeutic potential for synucleinopathies. A phase 1 first-in-human trial to investigate the safety, tolerability, pharmacokinetics, and pharmacodynamics of KM-819 in healthy subjects has been completed and revealed favorable safety, tolerability, and pharmacokinetics results [192] (NCT03022799). Recently, a phase 2 trial to further evaluate the safety and efficacy of KM-819 as a disease-modifying therapy to slow down the progression of PD was planned [193].

Lipid and fatty acid homeostases are crucial for neuronal functions in the brain, the second-most lipid-rich organ. A lipidomic analysis of α-synuclein neurotoxicity revealed that stearoyl-CoA desaturase (SCD) is essential for α-syn-induced neurotoxicity and that inhibiting SCD may be a novel therapeutic strategy [194–197]. A phase 1 study of SCD inhibitor YTX-7739 in healthy subjects has been completed and the result showed it was safe and well-tolerated. A following phase 1b safety and biomarker study has been conducted on PD patients, and the result reported no serious safety events, and a reduction in fatty acid desaturation in blood and CSF was observed. However, the FDA has placed a partial clinical hold on multi-dose clinical trials of YTX-7739 for some reason [198] (Trial NL9172).

A genetic relationship between *COQ2* mutations (*V393A* variant), which resulted in decreased production of Coenzyme Q10 (CoQ10) as an electron carrier in the mitochondrial respiratory chain and MSA-C type was exclusively established in Japanese population [25]. In addition, multiple mitochondrial dysregulations were observed in iPSC-derived dopaminergic neurons from MSA patients [199, 200]. In a report of a 3-year follow-up of high-dose ubiquinol supplementation in a case of familial MSA with *COQ2* mutations, the clinical rating scale scores remained stable, but mitochondrial oxidative metabolism improved [201]. A phase 2 multicenter study to evaluate efficacy and safety of high-dose ubiquinol (drug name: MSA-01) supplementation in MSA patients was completed (UMIN000031771). The results showed that high-dose ubiquinol was well-tolerated and led to a significantly smaller decline of UMSARS part 2 score compared with placebo in MSA patients, indicating orally administered ubiquinol have clinical benefits in patients with MSA [202].

During brain development, it is well-known that growth hormone promotes proliferation of neural precursors, neurogenesis, and gliogenesis, indicating its potential neuroprotective or neurotrophic effects [203]. A randomized, double-blinded, placebo-controlled pilot study in patients with MSA has been conducted and showed no treatment differences for any efficacy measures, but some trend improvements in terms of UMSARS total score and cardiovascular reflex autonomic testing. Maybe a large-scale trial and higher doses will be required for further studies [204].

Rasagiline is a monoamine oxidase type B (MAO-B) inhibitor that is approved for the symptomatic treatment of PD [205] and may have disease-modifying effect for PD, but because of the previous clinical studies have revealed that the treatment outcomes differ when different drug doses had been administered, further phase 3 trials are required [206] (NCT00256204). A promising preclinical study in the MSA transgenic mouse model with GCI pathology revealed improvement in motor deficits and significant reduction of neuronal loss [207]. However, a phase 2 trial to evaluate the efficacy, safety, and tolerability of Rasagiline in MSA-P patients failed, measured by UMSARS [208] (NCT00977665). In addition, another MAO-B inhibitor Safinamide was investigated to measure the treatment-emergent adverse events (TEAE) and serious adverse events (SAE) in MSA-P patients in the phase 2 stage (NCT03753763).

Boosting the levels of neurotrophic factors, such as brain-derived neurotrophic factor (BDNF) and glial cell line-derived neurotrophic factor (GDNF) in the brain, is an integral part of neuroprotective strategies. In animal research on the MSA transgenic mouse model, selective serotonin reuptake inhibitor Fluoxetine has been demonstrated to increase GDNF and BDNF [209, 210]. However, in a phase 2 trial in MSA patients failed to show improvement [211] (NCT01146548). Another trial on GDNF is focusing on gene therapy and currently recruiting to assess the incidence of TEAE and SAE within 3 years in a phase 1 trial of AAV2-GDNF in MSA (NCT04680065).

Some reported that the Insulin/IGF-1 signaling pathway contributed to the regulation of neuronal excitability, nerve cell metabolism, and cell survival, thereby supporting their neurotrophic function in a number of neurodegenerative diseases [212, 213]. Increased insulin and IGF-1 plasma concentrations in MSA patients and decreased IGF-1 brain levels in MSA transgenic mouse model supported the hypothesis that impaired insulin/IGF-1 signaling existing in MSA pathology [214–216]. A phase 2 study evaluating the efficacy of intranasal Insulin in patients with MSA (*n* = 1) and PD (*n* = 15) demonstrated an improvement based on UPDRS scale and the only MSA patient in this study received insulin-treatment remained symptom stable without disease progression [217] (NCT02064166).

Exendin-4, an FDA-approved antidiabetic glucagon-like pepdide-1 (GLP-1) analog, has been shown to protect the nigral dopaminergic neurons survival and reduce the α-synuclein load, but motor benefits in a MSA transgenic mouse model have not been observed [216]. A phase 2 randomized, open label study to evaluate the safety and efficacy of Exenatide (synthetic exendin-4) in patients with MSA is ongoing (NCT04431713).

Most recently, another neuroprotective target sphingosine-1-phosphate receptor 5 (S1P_5_) was focused and new clinical trial was initiated. S1P_5_ is predominantly expressed in nervous system and playing a role in neurodegenerative disorders [218]. A phase 2 study of S1P_5_ agonists ONO-2808 in patients with MSA is ongoing to assess the safety, tolerability, pharmacokinetics, pharmacodynamics, and potential efficacy as well (NCT05923866).

So far, the majority of completed clinical trials reported negative outcomes. The only clinical trial with positive outcomes is a trial of mesenchymal stem cells (MSC) treatment. As MSC is multipotent, it has been investigated as a potential neuroprotective or neurotrophic therapy [219–222]. Preclinical studies of MSC treatment in transgenic MSA mouse models supported that intravenously infused MSCs have a potent effect on immunomodulation and neuroprotection [223, 224]. The first clinical trial was an open-label, single-center study evaluating the feasibility and safety of therapy with autologous MSCs through consecutively intra-arterial and three repeated intravenous injections. The treatment group demonstrated significant improvement on UMSARS than the control group in all visits throughout the 12-month study period without serious adverse effects [225, 226]. However, the open-label design has been challenged. After that, a phase 2 trial of autologous MSCs in patients with MSA was completed and showed a smaller increase in total and part II UMSARS scores in MSA patients receiving autologous bone marrow derived MSCs (bmMSCs) via intra-arterial routes, indicating MSC therapy could delay the progression of neurological deficits in patients with MSA [227] (NCT00911365). However, because it was common to observe small ischemic lesions using magnetic resonance imaging (MRI) following intra-arterial infusion, safety concerns were raised to MSC therapies. Therefore, one recent phase 1 MSC clinical trial added the observation of small ischemic lesion as a safety measurement. The study reported that no ischemic lesions on diffusion-weighted images in any of the study participants, suggesting that a single intra-arterial administration of autologous bmMSCs is a safe and promising neuroprotective strategy in patients with MSA-C (NCT03265444). In addition, another ongoing long-term observational study also monitors the incidence of adverse events and the efficacy of subjects who participated in the above phase 1 trial to evaluate the safety and tolerability of autologous bone marrow derived MSCs in patients with MSA for up to 60 months after administration (NCT04495582).

MSCs derived from autologous fat (fMSCs) were also being investigated as a potential treatment to interfere MSA progression. A phase 1/2 open-label study evaluating the safety, tolerability, and efficacy of intrathecal injection of autologous MSCs in MSA patients was completed and revealed intrathecal MSCs was safe, well-tolerated, but associated with painful implantation at high doses [228] (NCT02315027). A phase 2 trial of intrathecally administered autologous fMSCs in patients with MSA is ongoing (NCT05167721).

In addition, a recent interventional phase 1/2a study is underway to assess the safety and efficacy of intrathecal administration of low-/high-dose allogenic human oral mucosa stem cells (hOMSCs) in MSA patients in early to moderate stage (NCT05698017).

Although the aforementioned clinical trials achieved their primary endpoints and represent a potentially significant advance in the treatment, the safety of intra-arterial injection remains controversial due to reports of micro-strokes and increased mortality.

## Discussion and outlook

MSA is a debilitating disease with poor prognosis. The difficulty in effectively managing the disease is not only due to the incomplete understanding of neuropathogenesis in terms of the interaction between neurons and glia, but also the lack of diagnostic methods, including specific biomarkers. The health-related quality of life (HR-QOL) of patients with MSA is significantly affected by aggressive disability due to motor and autonomic deficits and by nonmotor symptoms, especially depression. Regarding the measurement of clinical outcomes, a HR-QOL change scale could become a valuable tool and the basis for improved MSA outcome measurement associated with UMSARS, which could benefit future clinical trials of disease-modifying therapies [229–231].

### Genetic factors and future perspectives

MSA is largely regarded as a sporadic disease. In a recent [232] and this review, genetic factors, such as *SNCA, COQ2, C9orf72, LRRK2,* and *MAPT*, in MSA have been described thoroughly, even though there is still some controversy. In addition, some genetic factors seem to be region- or population-specific. Clearly, none of the genetic associations showed above is the direct causative factor of MSA. However, it is no doubt that genetic factors are the risk factors in MSA. To consolidate the genetic aspects in MSA, firstly, genome-wide association studies need to be conducted in different regions and populations. Importantly, it will be meaningful for identifying novel pathophysiological insights as well as innovative therapeutic interventions like personalized gene therapies if association between MSA risk and a series of genetic factors is identified.

### Open questions of MSA pathology and possible directions

Despite the tremendous progress regarding the underlying pathological mechanisms of MSA, further scientific research is needed to elucidate the interactions between neurons, oligodendrocytes, and other types of glia during the onset and progression of MSA.

MSA is grouped into neurodegenerative diseases (NDDs), in which eight hallmarks were presented in a recent review [233] as described in clinical trials. There are several critical unanswered questions in pathology of MSA. First is about the early stage of pathology, since it is quite likely pathologies underlying the disease start many years before clinical symptoms. Synaptic failure and dysfunction in many NDDs like AD and PD have been described as an early event before neurodegeneration [233]. Currently, an observational in vivo study is underway to investigate whether synaptic dysfunction will be an early critical alteration in MSA (NCT05121012). If so, it will provide new promising targets for MSA therapies. Second, all the NDDs share some pathological hallmarks and their commonalities may suggest that some strategies possibly have wider applications. On the other hand, at the final stage of NDDs, distinct brain regions and neuronal population were affected, indicating that disease-specific aspects should be considered in MSA. Lastly, as we know, GCIs is well-established hallmark in MSA as well as the central role in the diagnosis of MSA. So, an unanswered question is why α-synuclein accumulates in oligodendrocytes exclusively in MSA, but even not in other synucleinopathies. Hopefully, advances of high-sensitivity and -resolution approaches like single-cell sequencing may help further the unique transcriptome of MSA pathologies regarding the specific molecular alterations in oligodendrocytes in MSA and even across all the synucleinopathies, and then open new avenues for MSA therapeutic targets more specifically.

### Current picture of biomarkers

Biomarkers with higher sensitivity and specificity absolutely will benefit the early diagnosis of MSA, but also help monitor the prognosis and disease progression. In previous investigations of MSA, a number of biomarkers across several different sample sources were revealed as described earlier in this review. However, few biomarkers are put in the clinical practice. Among those biomarkers, total free and oligomeric α-synuclein were measured in an observational study to determine whether oligomeric α-synuclein level was elevated in MSA patients compared to controls (NCT01485549). In addition, another observational study (TRACK-MSA) in MSA patients is underway to define changes of CSF and plasma biomarkers, including α-synuclein, aggregated α-synuclein, Nfl and Tau/phosphorylated Tau in CSF, and plasma Nfl as well (NCT04450992). Taken together, the biomarkers above will be potentially diagnostic and prognostic biomarker if the clinical trials can provide some reliable profiles.

The lack of reliable biomarkers for early diagnosis and monitoring of disease progression complicates disease treatment. In fact, definitely, clear and specific pathological mechanisms may facilitate the exploration and promotion of novel biomarkers, and further the disease-modifying therapies. Although significant progress has been made on biomarker discovery for MSA, three recent observational clinical studies are underway to demonstrate the biomarker profiles across the synucleinopathies (NCT05453058, NCT05638815, NCT05699460). For future development of biomarkers, large multicenter observational studies are more convincing. Furthermore, high-throughput technologies like genomics, transcriptomics, proteomics will make the studies more reliable and efficient. Intriguingly, artificial intelligence also might assist this field. Indeed, high-sensitivity and -specificity, and non-invasiveness will be goals of new set of biomarkers.

### Possible therapeutic opportunities

Characteristic abnormal protein aggregation is a leading pathological hallmark of a variety of NDDs, including MSA and currently serves for diagnosis and disease-modifying therapeutic targets. Due to the central role of GCIs in MSA, integrating advances of gene therapies, targeting α-synuclein by gene therapy approach will be promising. Given FDA approved the ASO drug Nusinersen (Spinraza) for the treatment of SMA, and recently approved another ASO drug Casimersen (Amondys 45) for the treatment of DMD, ASOs have become a particularly attractive therapeutic strategy for MSA. Importantly, α-synuclein ASO has been showed to reverse the pathology in rodent PD models [126]. Taken together, reduction of α-synuclein production using ASOs may provide a disease-modifying therapy for MSA. Currently, a phase 1 study to assess the safety and tolerability of ASO of α-synuclein (ION464) administered intrathecally in MSA patients is ongoing (NCT04165486). Maybe it is the right time to consider the ASOs as a promising choice of treatment for CNS diseases like MSA due to conceptual simplicity, more selective action, less side effect, and wide distribution throughout the brain. However, there are still many challenges in terms of ASO delivery to the brain beyond the blood–brain barrier (BBB). In most of clinical trials and preclinical discovery of CNS diseases, intrathecal administration was employed and turned out it was a feasible and effective approach. But still, delivery via intrathecal way has a longer distance to travel to the brain, and also there is a high chance for dorsal root ganglion and spinal cord effects or damage. Last not the least, intrathecal administration is not a routine clinical procedure. Taken together, advances on ASOs delivery into the brain are required to be more specific, efficient and non-invasive. For the transfer of biomolecules across the BBB, transferrin receptor (TfR)-mediated delivery has significantly advanced this field [234]. It will be quite promising to attach the α-synuclein ASOs to the delivery cart like TfR-mediated one.

On the other hand, enhancing the degradation of pathological protein α-synuclein is also an attractive strategy, integrating current innovative technology PROTAC. As described in clinical trials, a peptide fusion containing α-synuclein binding domain and a short strong proteasome-targeting motif was able to bind to α-synuclein and direct it to the proteasome for degradation. There will be at least two kinds of challenges. The specific and efficient peptide must be developed in the first place. And delivery of this kind of biomolecules to the brain will face the same problem as ASO does. Therefore, the same strategy could be applied here.

Neuroinflammation, including microgliosis and astrogliosis, is a pathological hallmark of NDDs, including AD, PD and MSA as well. First, insights into the detrimental and protective roles of the different microglial populations will be important for effective therapeutic targeting in NDDs. Then, neuroinflammation is a cause of NDDs or just a result of other pathological hallmarks. Research will be needed to address above fundamental questions to further this direction. Interestingly, the nucleotide-binding domain, leucine-rich repeats-containing family, pyrin domain-containing-3 (NLRP3) inflammasome complex, comprising NLRP3, apoptotic speck protein containing a caspase recruitment domain (ASC), and cysteine aspartic acid protease 1 (Caspase 1), regulates microglial inflammation in several neurodegenerative diseases, including MSA. The study indicates that NLRP3 inflammasome is significantly upregulated and correlates with the neurodegenerative process in MSA [235], providing novel therapeutic strategies to target excessive activation of the inflammasome in MSA.

Last but not the least, neuroprotection is another fundamental theme in disease-modifying therapies of NDDs. The goal of neuroprotection treatment is to provide the brain with the necessary factors to support the neurons and to prevent neurodegenerative changes at the molecular level. First, some neurotrophic factors could be applied as described in clinical trials. Second, some core molecules at the key points of signaling pathways also can benefit as S1P_5_ did in clinical trials (NCT05923866). At last, across all clinical trials in MSA, the only clinical trial with positive outcomes is a trial of MSC treatment. The aforementioned clinical trials achieved their primary endpoints and represented a potentially significant advance in the treatment. Importantly, among all the MSC platforms, hOMSC retains properties of neural crest stem cells, due to its origins from the embryonic brain (neural crest). But the safety of intra-arterial injection for bmMSCs remains controversial due to reports of micro-strokes and increased mortality.

In conclusion, MSA is a mostly sporadic disease. Up to date, it still has been difficult to identify the disease initiators and to investigate the core pathological mechanisms. But a growing number of evidences showed that MSA has a genetic component. MSA is driven by combined defects like many other NDDs. Furthermore, integrating complicated pathological mechanisms, it points to the need for multi-targeted therapies. On the other hand, MSA belongs to the rare diseases and exhibited strong biological heterogeneity, which makes personalized therapeutic strategies promising. Therefore, designing combinatorial and personalized therapeutic strategies to effectively halt the MSA will be a promising and compelling perspective in future based on the better understanding the molecular mechanisms and development of biomarkers.

## Data Availability

No new data were created or analysed in this study. Data sharing is not applicable to this review article.
